# Genome-wide characterization of the hyperaccumulator *Sedum alfredii* F-box family under cadmium stress

**DOI:** 10.1038/s41598-021-82690-7

**Published:** 2021-02-04

**Authors:** Zhuang Zhang, Wenmin Qiu, Wen Liu, Xiaojiao Han, Longhua Wu, Miao Yu, Xuelong Qiu, Zhengquan He, HaiYing Li, Renying Zhuo

**Affiliations:** 1 Key Laboratory of Three Gorges Regional Plant Genetic and Germplasm Enhancement (CTGU)/Biotechnology Research Center, China Three Gorges University, Yichang, 443002 Hubei China; 2 State Key Laboratory of Tree Genetics and Breeding, Chinese Academy of Forestry, Beijing, China; 3 Key Laboratory of Tree Breeding of Zhejiang Province, The Research Institute of Subtropical Forestry, Chinese Academy of Forestry, Hangzhou, Zhejiang China; 4 Key Laboratory of Soil Environment and Pollution Remediation, Institute of Soil Science, Chinese Academy of Sciences, Nanjing, 210008 China; 5 Agricultural Technology Extension Center of Fuyang District, Hangzhou, Zhejiang China; 6 Institute of Virology and Biotechnology, Zhejiang Academy of Agricultural Sciences, Hangzhou, 310021 Zhejiang China

**Keywords:** Plant sciences, Plant stress responses, Abiotic

## Abstract

The F-box genes, which form one of the largest gene families in plants, are vital for plant growth, development and stress response. However, F-box gene family in *Sedum alfredii* remains unknown*.* Comprehensive studies addressing their function responding to cadmium stress is still limited. In the present study, 193 members of the F-box gene (SaFbox) family were identified, which were classified into nine subfamilies. Most of the SaFboxs had highly conserved domain and motif. Various functionally related cis-elements involved in plant growth regulation, stress and hormone responses were located in the upstream regions of SaFbox genes. RNA-sequencing and co-expression network analysis revealed that the identified SaFbox genes would be involved in Cd stress. Expression analysis of 16 hub genes confirmed their transcription level in different tissues. Four hub genes (*SaFbox40*, *SaFbox51*, *SaFbox136* and *SaFbox170*) were heterologously expressed in a Cd-sensitive yeast cell to assess their effects on Cd tolerance. The transgenic yeast cells carrying *SaFbox40*, *SaFbox51*, *SaFbox136*, or *SaFbox170* were more sensitive and accumulated more cadmium under Cd stress than empty vector transformed control cells. Our results performed a comprehensive analysis of Fboxs in *S. alfredii* and identified their potential roles in Cd stress response.

## Introduction

F-box protein family is one of the largest families in plant, actively involved in diverse function via protein degradation. Protein degradation is an important mechanism to regulate biological progress at post-translational level under various stresses in plant life cycle. Protein degradation, which is completed by UPS, is main proteolytic route in plant. In the late 1970s and early 1980s, the ubiquitin–proteasome system (UPS) was discovered in the laboratory of Avram Hirshko of the Israel Institute of Technology and Engineering^1^. The UPS degrades proteins and plays key regulatory roles in modulating plant responses to different biotic and abiotic stresses. This system is composed of an ubiquitin-activating enzyme (E1), an ubiquitin-conjugating enzyme (E2), and ubiquitin ligase (E3). A previous study confirmed that E3 was important for substrate recognition during ubiquitin-mediated protein degradation^2^. The SKP1–Cullin1–F-box (SCF) complex is the major type of E3 ligases. The F-box protein is responsible for substrate specificity, and the SCF complex determines the target substrate for UPS degradation by binding different F-box proteins^3,4^. Even so, several members of F-box proteins are involved in other biological processes not associated with the UPS.

The F-box proteins were named after a new protein domain detected in the cell cycle protein Cyclin F. The number of F-box genes in plants varies widely across species, with 156 genes in *Vitis vinifera*^5^, 226 genes in *Pyrus* spp^6^, 226 genes in chickpea^7^, 337 genes in *Populus trichocarpa*^8^, 687 genes in *Oryza sativa*^8^, and 694 genes in *Arabidopsis thaliana*. In most plants, additionally, the F-box proteins usually contain at least one conserved F-box domain, whose N-terminals bind to SKP1 and the target protein. Moreover, the conserved F-box domain generally comprises 40–60 amino acids. Some of the 694 *A. thaliana* F-box genes have been studied. For example, *UFO* (unusual floral organs), which was the first F-box protein-encoding gene identified in plants, had an important function related to floral meristem identity and floral organ development^9^. A study regarding *FOA1* suggested its role in abscisic acid signal that negatively regulated seed germination^10^. It has been reported that F-box (AT5G15710) exhibited differential expression patterns under cadmium stress^11^. F-box only protein 3 and auxin signaling F-box3 gene have been demonstrated that their expression were induced by Cd^12^.

Soil is critical for human survival and development, because it is an indispensable natural resource that influences agricultural productivity, ecological health, and sustainable development^13,14^. However, economic development has led to the exploitation of mineral resources and the extensive use of pesticides and fertilizers containing cadmium (Cd), resulting in worsening Cd pollution worldwide^15^. On April 17, 2014, the Ministry of Environmental Protection and the Ministry of Land and Resources of the People’s Republic of China jointly released “The National Soil Pollution Status Survey Bulletin”, which revealed that the overall soil exceeding the rate was 16.1% and the out-standard rate of the arable land reached 19.4%^16^. Additionally, Cd pollution is more harmful than other heavy metals. As a pollutant, Cd can be difficult to remove and is stable in soil because it is not easily degraded. After entering the soil, exogenous Cd will gradually accumulate and cause long-term damage to the soil and crops, ultimately endangering human health because it is transmitted through the food chain^17^. Thus, excessive Cd must be removed from soil^18^. By chance, *Sedum alfredii* was identified as a hyperaccumulator plant that could be able to absorb heavy metal (Pb, Zn, Cd) meanwhile normally growing. It is reported that it can accumulate Cd up to 1600 μg/g^19^. However, the mechanism underlying Cd accumulation and tolerance of *S. alfredii* is intricate. High through sequencing technology provides valuable information for the genome wide identification of stress-response gene family such as HSF^20^ , LRR-RLK^21^, and GDSL^22^. Although it has been reported that F-box involved in various abiotic stress responses^23^, but there is little knowledge about the molecular character of F-box under cadmium stress. The genome wide molecular characterization, expression pattern and function analysis of F-box gene family responses to the heavy metal cadmium (Cd) in the hyperaccumulator *S. alfredii* is yet to be studied in depth.

In present study, we identified F-box protein family from *S. alfredii* genome, analyzed biochemical characteristics of SaFbox including CDS length, number of amino acid, molecular weight, PI and amino acid length. Studying conserved domains and phylogenetic tree helped us know their phylogenetic relationships, investigating cis-acting elements was benefic for studying potential function of SaFboxs. In order to explore SaFboxs’ regulatory network, we screened hub genes and the genes it potentially regulated. The expression profiles of S. *alfredii* F-box genes were investigated via RNA-seq data. Furthermore, the expression abundance of SaFbox hub genes were confirmed by quantitative real-time qRT-PCR. For testing the function of SaFbox, we investigate tolerance of transgenic yeast expressing *SaFbox40*, *SaFbox51*, *SaFbox136*, *SaFbox170* and empty vector treated with Cd. The results presented in this study provided useful information on F-box proteins in *S. alfredii* for future functional studies, particularly the proteins that may have important functions in response to Cd stress.

## Materials and methods

### Identification of *Sedum alfredii* SaFbox proteins

The hidden Markov model (HMM) profiles of F-box (PF00646), F-box-like (PF12937), and F-box-like 2 (PF13013) downloaded from the Pfam database (http://pfam.xfam.org/) were used as queries to screen genomic data. All putative *S. alfredii* F-box proteins were confirmed with INTERPRO. Basic physical and chemical properties of proteins, including the number of amino acids, isoelectric points, and molecular weights, were predicted with ExPASy (https://web.expasy.org/protparam/).

### Phylogenetic analyses

The SaFbox protein sequences were aligned with ClustalX in MEGA5 using GONNET as the protein weight matrix, with a gap opening penalty of 10 and a gap extension penalty of 0.1. Phylogenetic tree was constructed according to the neighbor-joining method, with the following parameters: test of phylogeny = bootstrap method; number of bootstrap replications = 1000; and gaps/missing data treatment = complete deletion. The iTOL program was used to modify the phylogenetic trees^24^.

### Conserved motif and amino acid residue analyses

The SaFbox motifs were predicted with the following parameters of an online tool (http://meme-suite.org/tools/meme): motif discovery = classic mode; maximum number of motifs = 100; number of repetitions = 0 or 1 occurrence per sequence; and optimum motif width = 6–100 residues. The SaFbox sequences were aligned with the ClustalX software. The same number of amino acid residues in the conserved regions was output. Sequence logos based on the patterns in the multiple sequence alignment were generated with the WebLogo online program (http://weblogo.threeplusone.com/create.cgi).

### Chromosomal locations, Promoter cis-acting element

The locating information of all SaFbox were obtained from GFF3 and visualized by software Mapinspect^25^.The cis-acting elements in the promoters 2.0 kb upstream of the SaFbox genes were analyzed with the PlantCARE online program (http://bioinformatics.psb.ugent.be/webtools/plantcare/html/). Part of cis-acting elements about stress were visualized with TBtools^26^.

### Co-expression network analysis

A weighted gene co-expression network analysis was used to construct transcriptional networks based on the profiles of differentially expressed genes responsive to Cd stress as previously described^27^. Pearson’s correlation coefficients for the fragments per kilobase of transcript per million fragments mapped (FPKM) values for each gene pair were calculated with the R program, with the correlation coefficient threshold set to 0.40. We screened the SaFbox family members and identified the hub genes in the co-expression network. The relevant edges were classified according to their annotations, and we further analyzed their associations with hub genes. Finally, the co-expression regulatory network was visualized with Cytoscape (version 3.6.1).

### Heterologous gene expression in yeast

Three *S. alfredii* hub genes (*SaFbox40*, *SaFbox136*, and *SaFbox170*) were isolated and ligated into the yeast overexpression vector (pYes2.0) via homologous recombination. The recombinant plasmids and empty vector (control) were transformed into Δ*ycf1* yeast strain cells. Transgenic yeast cells were cultivated in liquid nutrient medium with synthetic galactose-uracil (SG-U) until the optical density (600 nm) reached 0.8–1.0, after which the cells were spotted on agar-solidified SG-U medium containing 0 or 50 µM CdCl_2_. The SG-U liquid cultures were diluted (0, 1/10, 1/100, 1/1000, 1/10,000, and 1/100,000) and then incubated at 28 °C for 3 days. Additionally, the relative growth of transformants was determined by measuring the optical density at 600 nm at 12 h intervals. The Cadmium concentration of transgenic yeast were detected when they were cultured at logarithmic phase under 30 μM/L cadmium stress.

### Plant materials and stress treatments

*Sedum alfredii* plants collected from an old Pb/Zn mining area in Quzhou city were asexually reproduced in a hydroponics system at 25 °C with a 16-h light/8-h dark cycle. The seeding which were vegetative propagation form Quzhou city grown in a half-strength Hoagland solution for about four weeks, after which the roots were treated with 100 µM CdCl_2_. The roots, stems and leaves were sampled at 0, 4, 12, 24, 36, and 48 h, with 3 biological replicates per sample. All samples were stored at − 80 °C until analyzed.

### Total RNA isolation and expression analysis

Total RNA was extracted from the collected plant tissues with a commercial RNA extraction kit (NORGEN, Thorold, Canada). The RNA samples were treated with RNase-free DNase I (New England BioLabs, Ipswich, MA, USA) to eliminate any residual genomic DNA. The RNA concentration and quality were determined with the NanoDrop 2000 spectrophotometer (Thermo, Wilmington, DE, USA). Finally, the RNA served as the template for generating first-strand cDNA with the PrimeScript RT Master Mix (TaKaRa, Dalian, China). The cDNA was stored at − 80 °C until analyzed by qRT-PCR.

A qRT-PCR assay was conducted with the SYBR Premix Ex Taq reagent (TaKaRa) and the 7300 Real-Time PCR System (Applied Biosystems, Foster City, CA, USA). The relative expression level of each SaFbox gene was calculated based on the 2^−ΔΔCt^ method^28^. The *SaUBC9* gene was used as an endogenous reference control to normalize the threshold values (Ct) of the SaFbox genes^29^.

## Results

### Identification and phylogenetic analysis of F-box genes in *S. alfredii*

A preliminary screening identified 285 candidate F-box family members by HMMER3.0 software^30^. To eliminate redundant sequences, the candidates were verified with SMART and InterPro as well as BLAST searches^31^. Finally, a total of 193 F-box genes were identified (Table S1). The CDS length of F-box ranged from 249 bp (SaFbox190) to 2898 bp (SaFbox99). Protein length which they encode ranged from 83 to 963 amino acids (aa) with molecular weights (9.17 to 110.6 kDa) and isoelectric points (4.19 to 10.69), all of which are listed in Table S2.

**Figure 1:**
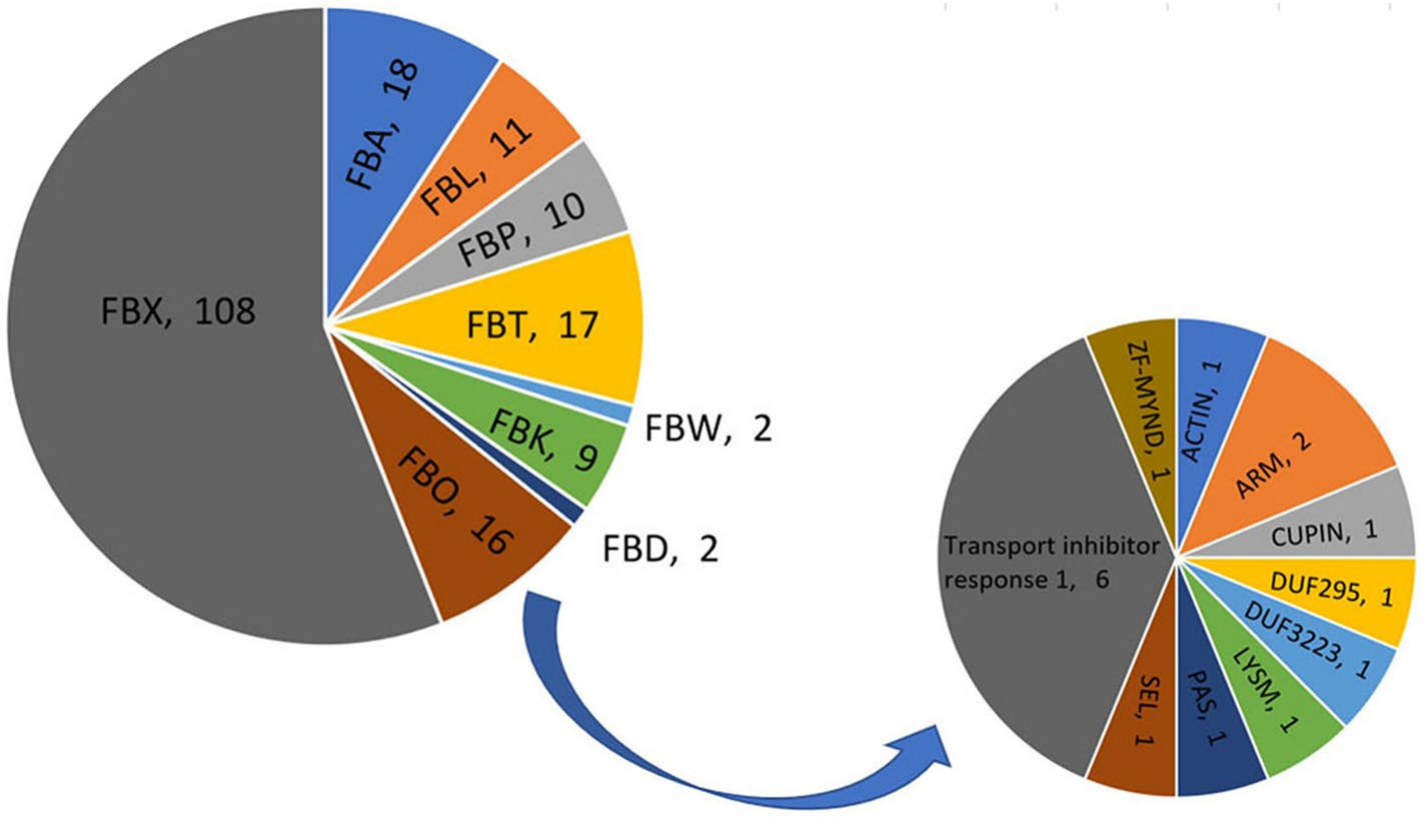
Classification of F-box proteins in *Sedum alfredii* according to C-terminal domains. Pie chart was constructed by software EXCEL; Different color display different subfamily.

Based on the encoded conserved C-terminal domain, the genes were classified into nine subfamilies (Fig. 1). The members of the largest subfamily, FBX (108), only contain an F-box domain. The other subfamilies and their domains were as follows: FBA (18) with an F-box-associated domain, FBT (17) with the TUBBY domain, FBP (10) with the PP2 domain, FBK (9) with the kelch domain, FBD (2) with the FBD domain, FBW (2) with the WD40 domain, and FBO (16) with other domains, including SEL, JMJC, and ACTIN (Table S3)^32,33^.

**Figure 2:**
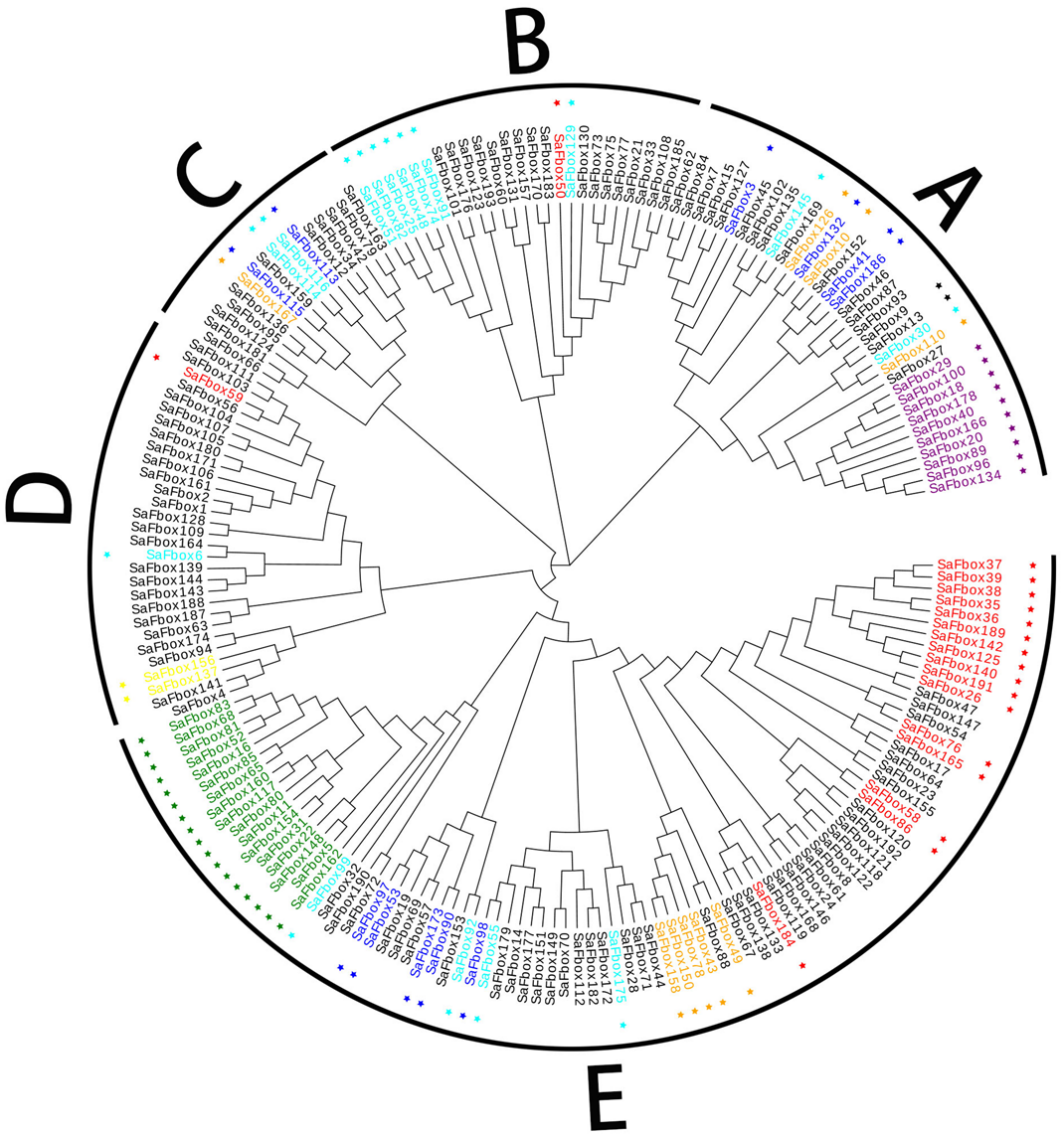
Analysis of phylogenetic relationships. The full amino acid sequences of 193 SaFbox proteins were aligned and a phylogenetic tree was constructed with MEGA7.0. Clades were divided into six groups (A–E). Different subfamilies are presented in different colors. Blue represents FBL subfamily, cyan (FBO), red (FBA), purple (FBP), orange (FBK), yellow (FBK), green (FBT), dark (FBX) and light green (FBW).

To characterize phylogenetic relationships of SaFbox proteins, we constructed a phylogenetic tree according to the neighbor-joining method. The SaFbox proteins were classified into five subgroups (A–E) based on the alignment of full sequences and the evolutionary distances (Fig. 2). Additionally, the phylogenetic tree was divided into five distinct branches based on the full sequence. The large FBX subfamily formed its own branch. Interestingly, some of the SaFbox proteins with the same C-terminal domain were clustered in different branches. For example, *SaFbox167*, which has a C-terminal kelch domain, was not clustered in Group C.

### Analyses of conserved amino acid residue and chromosomal Locations

**Figure 3:**
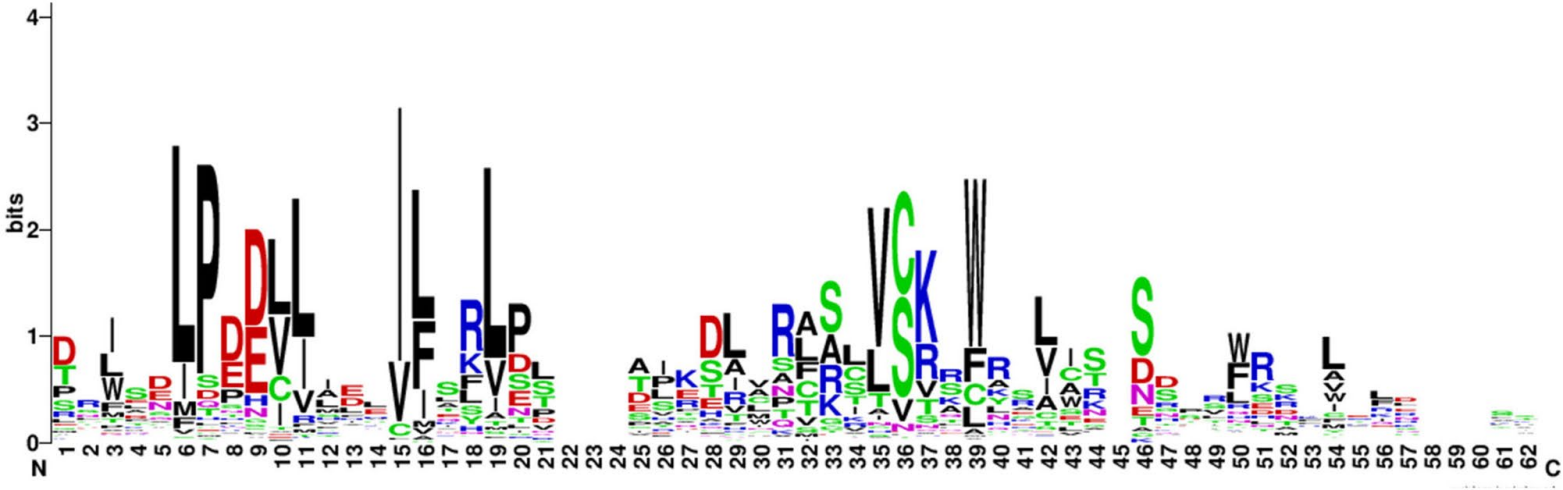
The conservation of SaFbox amino acid residues were analyzed. The numbers on the x-axis indicate amino acid positions. The size of the amino acid on the y-axis indicates the number of times the amino acid appears in a particular position.

The conserved N-terminal F-box motif comprises 40–60 amino acids. To study the F-box DNA binding residues in *S. alfredii*, all F-box’ domains were aligned in a WebLogo sequence logo^34^ (Fig. 3), which revealed that Leu-6 (68.9%), Pro-6 (73.5%), Val-15 (73.57%), Leu-19 (62.7%), and Trp-39 (62.9%) are highly conserved amino acid residues. These results are consistent with those of previous studies^34,35^, and suggest that these conserved residues may bind specifically to SKP to mediate important biological functions. This information may be useful for further investigating SaFbox functions.

**Figure 4:**
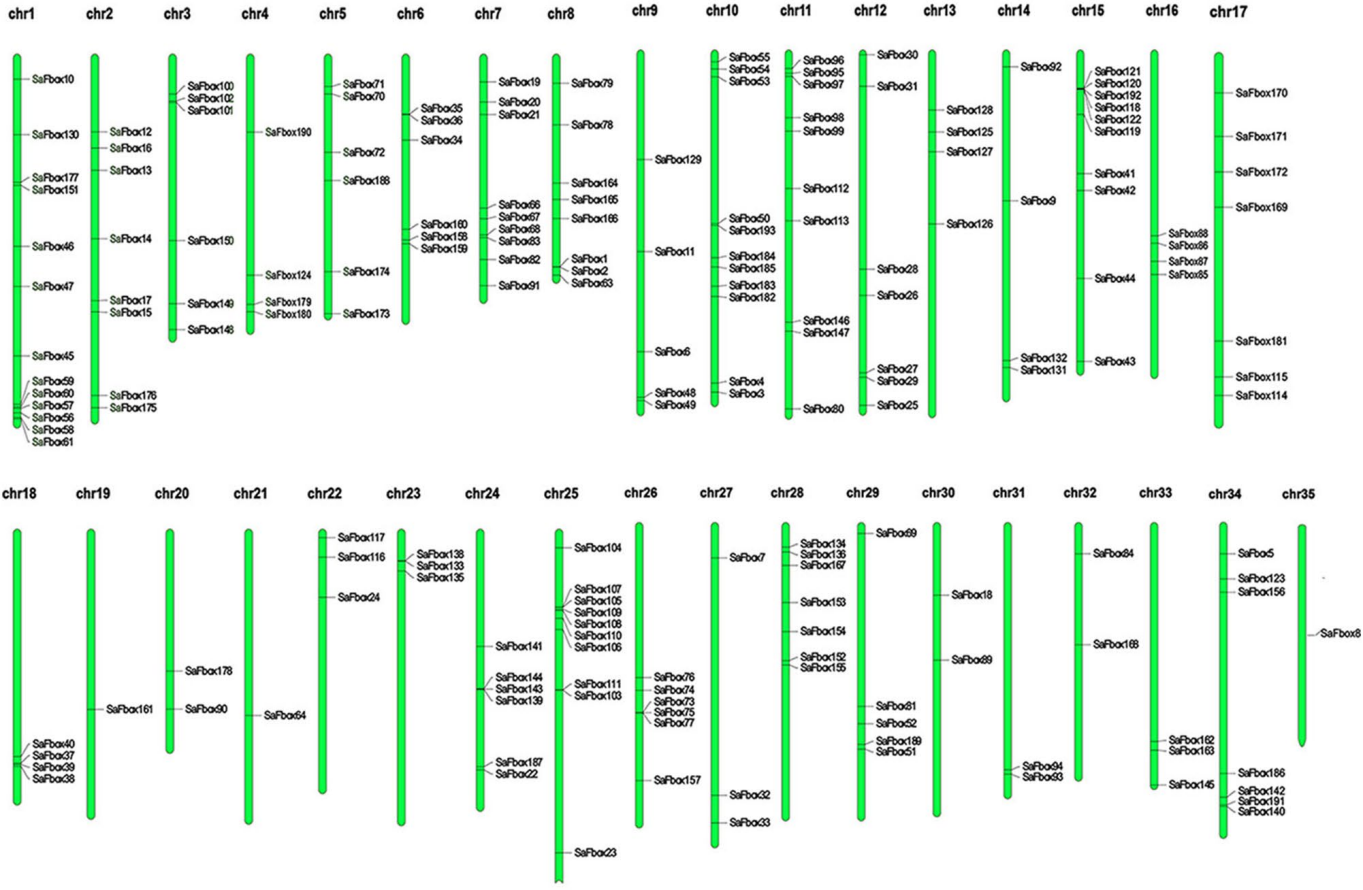
Chromosomal locations of SaFbox genes*.* A total of 193 SaFbox were distributed in 35 chromosomes. The chromosomes are drawn to scale and the chromosome numbers are indicated at the top of each. The gene names are shown on the right side of each chromosome corresponding to the position of each gene. Blue boxes represent different Chromosomal.

To investigate SaFbox distribution on Chromosomal, we got the chromosomal location of SaFbox gene from *S. alfredii* genomic database. A genomic analysis localized the SaFbox genes on specific chromosomes (Fig. 4). Specifically, most SaFbox genes were distributed on different chromosomes, with most located on chromosomes 1, 3, 6, 15, 18, and 25. The largest number of SaFbox locates on chromosomes 1 which contains 13 family members. Chromosomes 21 and 35 contains the fewest family member (1). *SaFbox62, SaFbox 65, SaFbox 137* are not mapped to the chromosome of the genome.

### Analysis of cis-acting elements in SaFbox promoters

**Figure 5:**
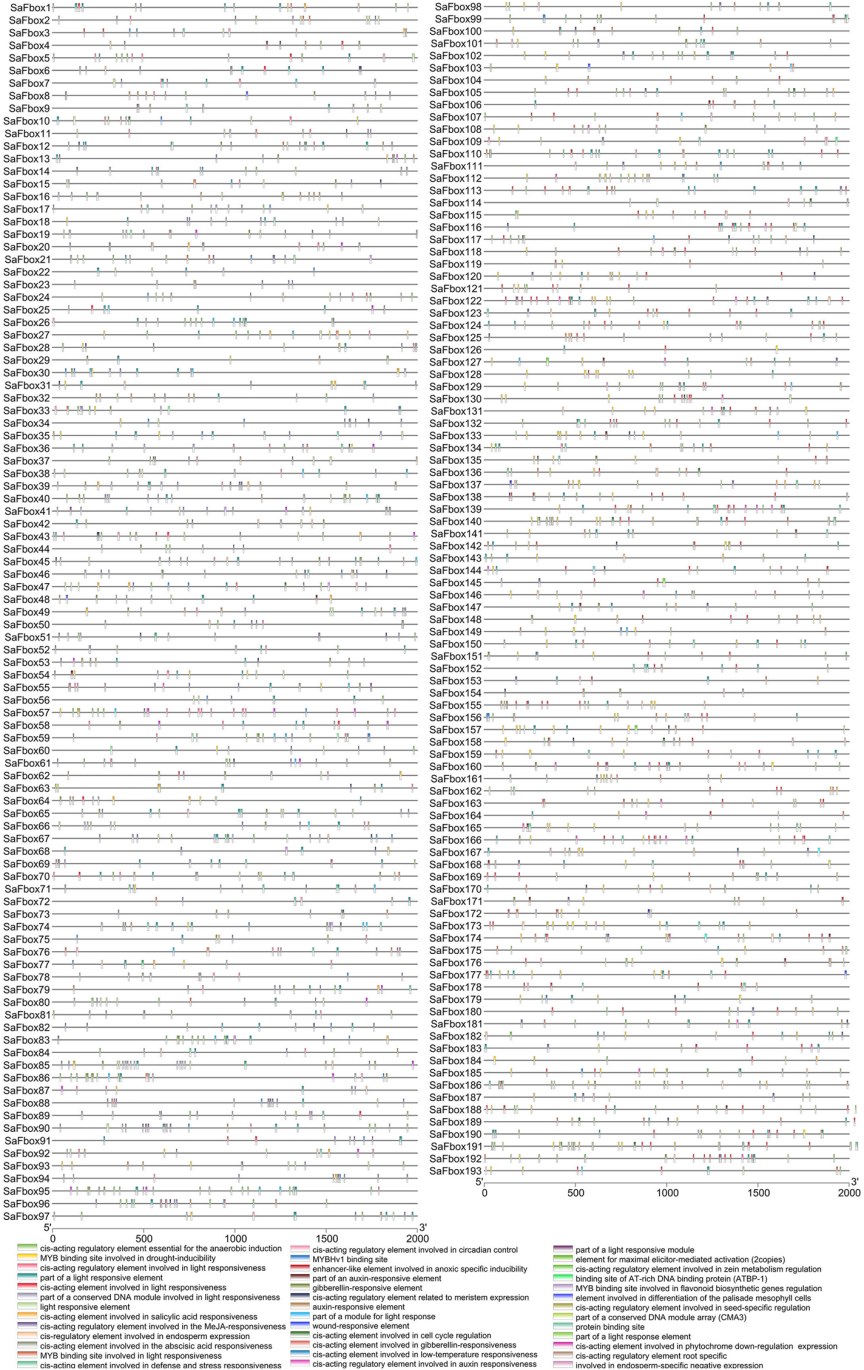
The distribution of related cis-acting elements from the promoters of SaFbox genes. Different boxes represent different cis-acting elements.

2 kb upstream flanks of SaFbox genes were retrieved to detected the cis-acting elements in the PlantCARE database (Table S4)^36^. We identified many cis-acting elements involved in various pathways, including the TC-rich element related with defense and stress signaling, the GC motif related with anoxic signaling, and the MBS related with drought signaling^16^. Cis-element about biotic stress also were found including responding to ABA, SA, GA, MeJA and Auxin in SaFbox’ promoter sequence. The related cis-acting elements and their distribution are presented in Fig. 5.

### Co-expression network

**Figure 6:**
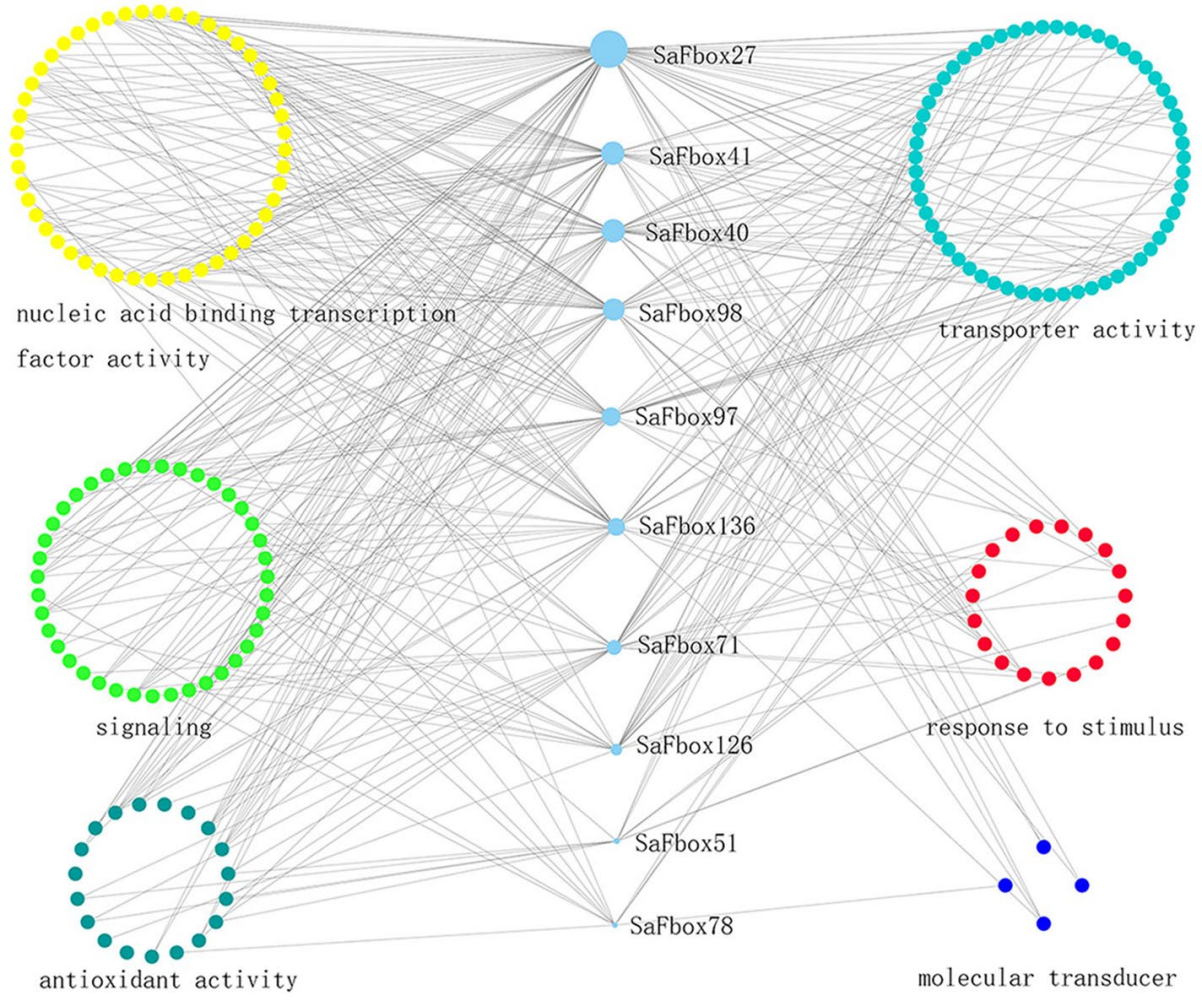
Co-expression network of SaFbox genes. Nodes represent individual genes and edges indicate significant co-expression between genes. Genes involved in the same biological process are grouped together distinguished by different colors.

To evaluate the importance of hub genes in gene regulatory networks, we further analyzed the transcriptome data, resulting in the identification of 20 hub genes (Table S5). A co-expression network (Fig. 6) was developed with Cytoscape to present the 20 hub genes with edge genes^37,38^. The major categories included nucleic acid binding transcriptional factor activity, transporter activity, signaling, response to stimulus, antioxidant activity and molecular transducer, implying the vital function of SaFboxs in response to Cd stress . With the most edge genes in the module, *SaFbox27* may play important roles under Cd stress. Among them, several hub genes such SaFbox40, SaFbox136, SaFbox170 seemed to be linked with different specific genes for various biological processes, suggesting that they might have different functions.

### Expression pattern of SaFbox under Cd stress

**Figure 7:**
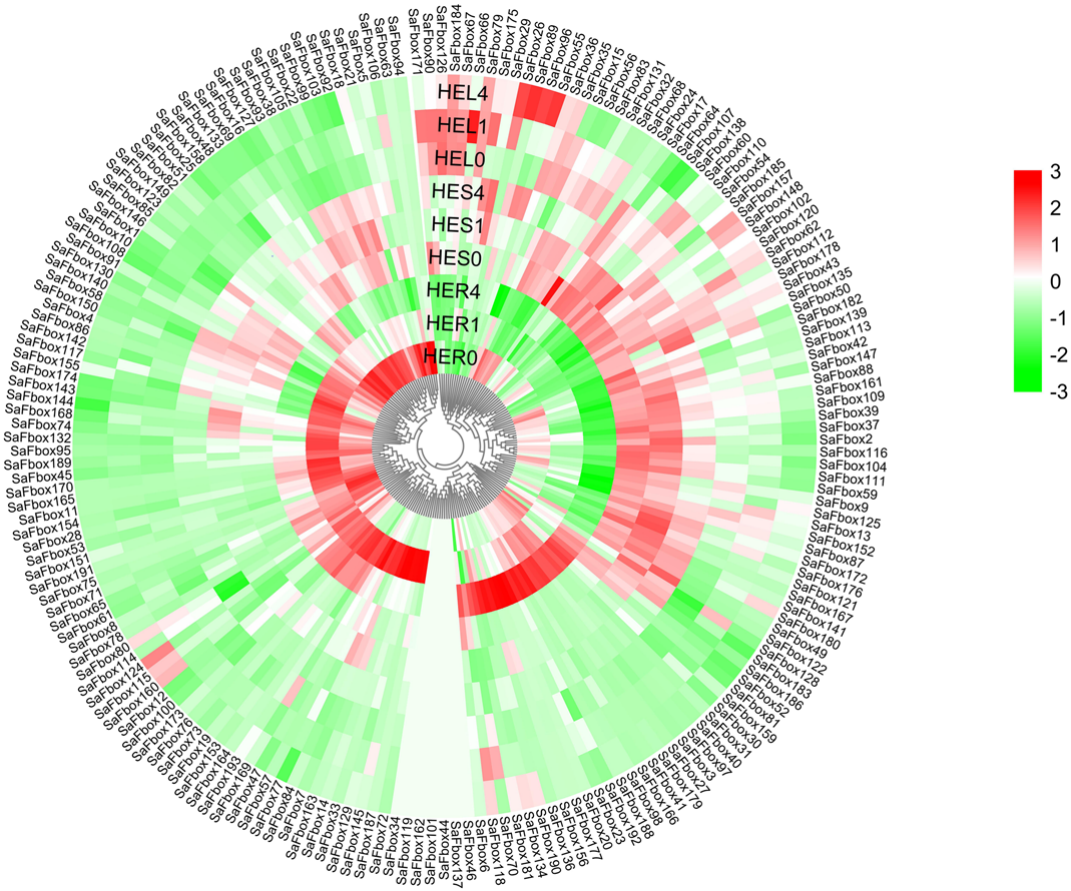
SaFbox expression patterns in various tissues response to Cd stress. The normalized RPKM values of the expressed genes were log2-transformed; Color scale at the left represents the expression levels authorized by Z-Score analysis.

**Figure 8:**
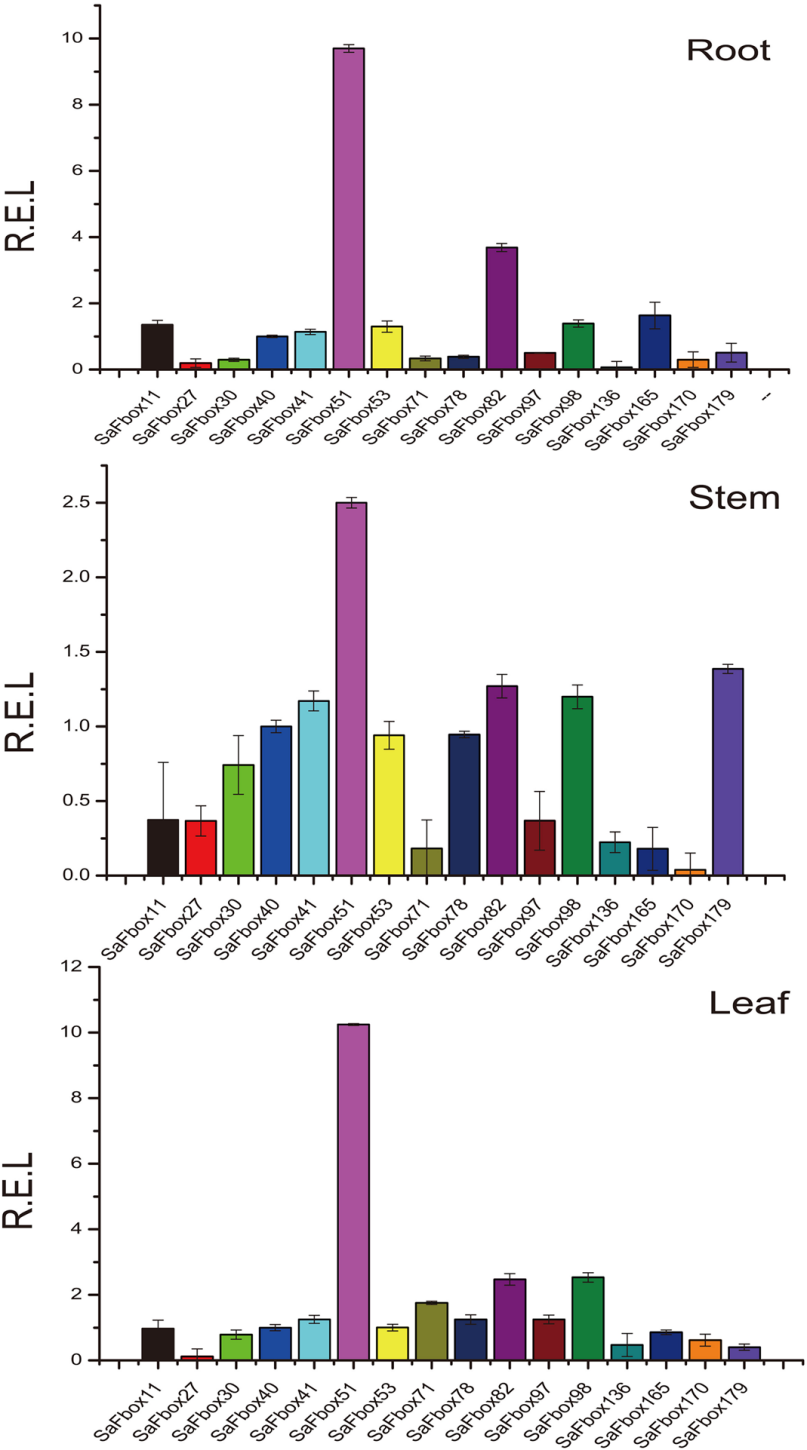
Expression profiles of 16 hub genes at different tissues under normal growth condition. (**A**) Root; (**B**) Stem; (**C**) Leaf. The gene expression levels relative to *SaFbox40* were shown. Bars indicate means ± standard deviations (SDs) of at least three independent biological replicates.

Uncovering expression levels of SaFbox, we analyzed SaFbox expression pattern by transcriptome in different tissues and stages under Cd stress, the transcriptome data of SaFbox display in Table S6 that visualized in Fig. 7. We found that most SaFboxs respond to Cd, except SaFbox34, *SaFbox119*, *SaFbox162*, *SaFbox101*, *SaFbox44*, *SaFbox137* and *SaFbox46*. Significantly, different expression profiles of SaFbox was found in root and stem under Cd. However, expression changes of SaFbox is not obvious in leaf. A few SaFbox’ expression is high in leaf such as *SaFbox55*, *SaFbox66*, *SaFbox26*, *SaFbox89*, and *SaFbox96*. About quarter of SaFbox respond to Cd in stem. About half of SaFbox respond to Cd at different states in root. Among them, some hub genes were up regulated under Cd stress. The expression of *SaFbox136* is about 13 folds than normal growth after 4d under Cd stress. The tissue specific expression of *SaFbox40* is high at any stages in root as well as SaFbox170 under Cd stress. These results provide important information for investigating the effects of Cd stress on SaFbox genes. Meanwhile, we analyzed the hub gene expression of SaFbox under normal growth by qRT-PCR (Fig. 8). Only one gene (*SaFbox51*) is high expression in all tissues (root, stem and leaf). Most hub genes showed a medium expression level throughout the entire plants under normal growth.

### Hub gene expression in yeast

**Figure 9:**
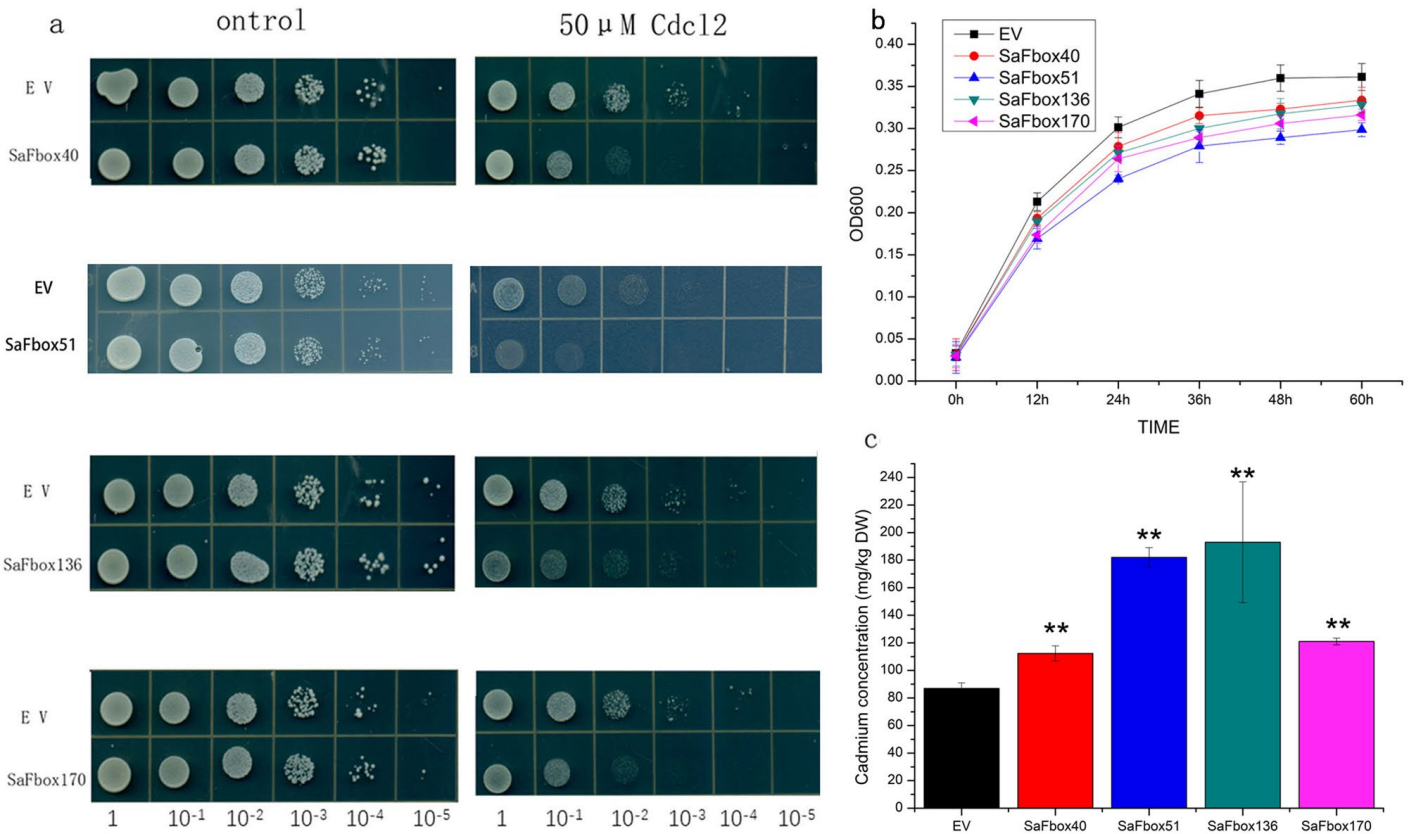
Effects of the overexpression of *Sedum alfredii SaFbox40*, *SaFbox136*, and *SaFbox170* genes in yeast. (**a**) The growth of Δycf1 yeast cells carrying the pYes2.0 empty vector (control) is compared with that of cells carrying recombinant plasmids with *SaFbox40*, *SaFbox136*, *or SaFbox170*. (**b**) Growth curves of transgenic yeast with SaFboxs or empty vector in SG-U liquid medium treated with Cd. (**c**) Cadmium accumulation of transgenic yeast were detected at logarithmic phase under 30 μM/L CdCl_2_. Bars indicate means ± standard deviations (SDs) of at least three independent biological replicates. Two asterisks indicate a significant difference at *p* < 0:01 compared with an empty vector.

We choose three hub genes which expression raises rapidly under Cd stress by above result. Based on the above-mentioned results, three hub genes (*SaFbox40*, *SaFbox136*, and *SaFbox170*) were overexpressed in yeast cells exposed to Cd stress. The growth curves indicated that all cells carrying the SaFbox genes were more sensitive to Cd stress than the control cells carrying the empty vector. The result display in Fig. 9. Thus, overexpressing *SaFbox40*, *SaFbox51*, *SaFbox136*, and *SaFbox170* reduced cadmium tolerance in yeast. Meanwhile, the Cd concentrations in yeast transformants were measured. As showed in Fig. 9c, expression of *SaFbox40*, *SaFbox51*, *SaFbox136*, or *SaFbox170* significantly increased the Cd content in yeast cells. The Cd concentration in the yeast transformant were higher than that of the yeast cells containing empty vector. Silencing these genes may enhance Cd tolerance in yeast. However, it is needs further verification that overexpressing these genes is the same function in *S. alfredii* with yeast*.* The mechanism underlying the effects of these genes is unknown and will be thoroughly investigated in future studies.

## Discussion

F-box family has been identified in different species, such as *Saccharomyces cerevisiae* (14)^32^, human (26)^39^, Drosophila (22)^40^. However, animal F-box family number is far less than plant, which means that F-box plays vital roles in plant. F-box family number has relatively large differences in plant. Previous result shows that F-box family were identified in *A. thaliana* (694), *O. sativa* (687), *Z. mays* (359), *C. arietinum* (285), *Pyrus spp* (226). It has been reported that F-box gene numbers are expanded in annuals herbs relative to perennial wood^8^. Analyzing F-box family in 18 species, the F-box family number is not relative with species and genome size^32^. The plentiful F-box family number may be caused by gene duplication in evolution. In order to properly process and respond to complex environmental changes, F-box gene may be lose or mutated in long period of evolution, which caused quantity variance in different species. Even though F-box is one of large protein family in plant, most of the F-box’s function remain unknown. In this study, we identify 193 SaFbox members, which is lesser than other species in plant that may be caused by specific expansion and functional diversification.

193 SaFboxs were classified into nine subfamily base on full protein sequence, among which the most abundant SaFboxs belonged to the FBX subfamily with 108 members, consistent with the results of previous studies^32^. However, the second largest subfamily, FBA, comprises 17 members, which differs from the findings for other species^41,42^. These suggest that FBA members may carry functions related to *S. alfredii*–specific cellular precess. The diversity in their C-terminal domains suggest the SaFbox proteins have diverse roles involved in plant growth and development. Thus, further functional analysis should be attempted to unveil their functions particularly in the Cd response. Besides, previous researches regarding F-box proteins focused primarily on the UPS pathway, relatively little is known about F-box functions in other pathways.

To further investigate the potential regulatory networks of SaFbox, we analyzed SaFbox’ promoter sequence. We found large number of cis-elements, which are related with circadian control, biotic and abiotic stress. A large number of cis-elements are relative with light. *EID1*, which contains F-box protein, has been proved to involve in light signaling. SaFboxs that are highly homologous with EID1 may respond to light in the same pathways. Part of cis-elements is relative with biotic stress including responding to salicylic acid, MeJA, abscisic acid, auxin and gibberellin. F-boxs that are involved in biotic stress also have been demonstrated. Analyzing cis-elements shows that *SaFbox48*, *SaFbox49*, *SaFbox111*, *SaFbox137*, *SaFbox187* contain five cis-elements about biotic stress (ABA, SA, GA, MeJA and Auxin). MBS (drought-inducibility), LTR (low-temperature), TC-rich (defense and stress) elements were unique in the promoter sequences of three SaFbox’s (*SaFbox17*, *SaFbox34* and *SaFbox174*). These results show that SaFboxs were involved in various biotic and abiotic stress.

Numerous F-boxs have been reported that it was involved in various abiotic stresses. however, limited studies on the SaF-box genes that respond to abiotic stresses especially for Cd have been conducted. Here we used previous RNA-seq data to study the expression pattern of SaF-boxs in different tissues under Cd stress. About Three-quarter SaFboxs were identified as differentially expressed genes in response to Cd stress in roots. The expression of Some SaFboxs (*SaFbox55*, *SaFbox66*, *SaFbox89*, *SaFbox96*) are upregulated after Cd stress for 4 day in leaf and stem, but their expression levels were relatively low in root under normal growth. Specific differential expression patterns indicated that these GmFBXs would play diverse functions in different tissues.

Three hub genes (*SaFbox40*, *SaFbox136*, and *SaFbox170*) may be involved in plant responses to Cd stress. *AtPP2* has been reported that it can respond to drought and salt^43,44^. As a homologous gene to *AtPP2*, *SaFbox40* might have similar roles. It is still unknown whether *AtPP2* responds to drought and salt via the Ub/26S proteasome pathway. Nevertheless, *SaFbox40* can respond to Cd, it is estimated that *SaFbox40* may respond to Cd by the same pathways as *AtPP2*. *SaFbox136*, *SaFbox170* only contains an F-box domain without others, it is logical to speculate that they do not respond to cadmium stresses by UPS pathways. The yeast complementation assay revealed that the hub genes increased the Cd sensitivity in mutant strain. These yeasts expressing hub genes were able to accumulate more Cd content than than that containing empty vector. Cadmium stress inhibited growth of transgenic yeast more seriously than empty vector, however how and where they function are unclear. Accordingly, silencing these genes may help elucidate the mechanism underlying their functions. Although some basic details of SaFbox genes were revealed in this study, the specific functions of the encoded proteins need to be investigated further.

## Conclusions

In our study, 193 F-box genes were identified in a genome-wide analysis of *S. alfredii* and then classified into nine subfamilies based on the encoded conserved C-terminal domains. A motif analysis of SaFbox proteins detected five conserved amino acid positions Leu-6 (68.9%), Pro-6 (73.5%), Val-15 (73.57%), Leu-19 (62.7%), and Trp-39 (62.9%). An examination of the SaFbox promoter elements suggested that SaFbox proteins might contribute to various physiological and molecular processes. The observed SaFbox expression levels in diverse tissues revealed that some genes play important roles in response to Cd stress, including *SaFbox40*, *SaFbox51*, *SaFbox136*, and *SaFbox170*. The data presented herein may provide the foundation for improving the remediation of soils contaminated with heavy metals.

## Supplementary Information

Supplementary Information.

Supplementary Tables.

## References

[1] Sadanandom A, Bailey M, Ewan R, Lee J, Nelis S (2012). The ubiquitin-proteasome system: central modifier of plant signalling. New Phytol..

[2] Stone SL (2014). The role of ubiquitin and the 26S proteasome in plant abiotic stress signaling. Front. Plant Sci..

[3] Huo DY (2014). Identification, classification, and drought response of F-box gene family in foxtail millet. Acta Agron. Sin..

[4] Wang GM (2016). F-box genes: genome-wide expansion, evolution and their contribution to pollen growth in pear (Pyrus bretschneideri). Plant Sci. Int. J. Exp. Plant Biol..

[5] Yang X (2008). The F-Box gene family is expanded in herbaceous annual plants relative to woody perennial plants. Plant Physiol..

[6] Guo-Ming W (2016). F-box genes: genome-wide expansion, evolution and their contribution to pollen growth in pear (Pyrus bretschneideri). Plant Sci..

[7] Gupta S, Garg V, Kant C, Bhatia S (2015). Genome-wide survey and expression analysis of F-box genes in chickpea. BMC Genom..

[8] Yang X (2008). F-box gene family is expanded in herbaceous annual plants Arabidopsis and rice relative to woody perennial plant Populus. Plant Physiol..

[9] Samach AKlenz JE, Kohalmi SE, Risseeuw E, Haughn GW, Crosby WL (2010). The unusual floral organs gene of Arabidopsis thaliana is an F-box protein required for normal patterning and growth in the floral meristem. Plant J..

[10] Peng J (2012). Arabidopsis F-box gene FOA1 involved in ABA signaling. Sci. China Life Sci..

[11] Remans T (2008). Normalisation of real-time RT-PCR gene expression measurements in Arabidopsis thaliana exposed to increased metal concentrations. Planta.

[12] Zhao SZ, Jian BS, Zhi MY (2012). Genome-wide identification of microRNAs and their targets in response to cadmium. J. Exp. Bot..

[13] Zhong L (2019). Phytoremediation potential of Pterocypsela laciniata as a cadmium hyperaccumulator. Environ. Sci. Pollut. Res. Int..

[14] Yang H, Zhang H, Liu Y, Xu Y, Dai J (2017). Characteristics and its assessment of heavy metal content in soil and rice with different repair methods. Trans. Chin. Soc. Agric. Eng..

[15] Luo Y (2019). Study on the repair of heavy metal contaminated soil. IOP Conf. Ser. Earth Environ. Sci..

[16] Li H (2019). Identification and expression analysis of the GDSL esterase/lipase family genes, and the characterization of SaGLIP8 in Sedum alfredii Hance under cadmium stress. PeerJ.

[17] Xu F (2017). Assessment of heavy metal contamination in urban river sediments in the Jiaozhou Bay catchment, Qingdao, China. CATENA.

[18] Fang A, Dong J, An Y (2019). Distribution characteristics and pollution assessment of soil heavy metals under different land-use types in Xuzhou City, China. Sustainability.

[19] Deng DM (2007). Zinc and cadmium accumulation and tolerance in populations of Sedum alfredii. Environ. Pollut..

[20] Chen SS, Jiang J, Han XJ, Zhang YX, Zhuo RY (2018). Identification, expression analysis of the Hsf family, and characterization of class A4 in Sedum alfredii Hance under Cadmium stress. Int. J. Mol. Ences.

[21] He X, Feng T, Zhang D, Zhuo R, Liu M (2019). Identification and comprehensive analysis of the characteristics and roles of leucine-rich repeat receptor-like protein kinase (LRR-RLK) genes in Sedum alfredii Hance responding to cadmium stress. Ecotoxicol. Environ. Saf..

[22] He L (2019). Identification and expression analysis of the GDSL esterase/lipase family genes, and the characterization of SaGLIP8 in Sedum alfredii Hance under cadmium stress. PeerJ.

[23] Sheng Z (2012). Genome-wide identification of Brassica napus microRNAs and their targets in response to cadmium. J. Exp. Bot..

[24] Letunic I, Bork P (2016). Interactive tree of life (iTOL) v3: an online tool for the display and annotation of phylogenetic and other trees. Nucl. Acids Res..

[25] Zhang SP, Liu MM, Miao H, Zhang SQ, Gu XF (2012). Chromosomal mapping and QTL analysis of resistance to Downy Mildew in Cucumis sativus. Plant Dis..

[26] Chen C, Chen H, Zhang Y, Thomas HR, Xia R (2020). TBtools: an integrative toolkit developed for interactive analyses of big biological data. Mol. Plant.

[27] Han X (2016). Integration of small RNAs, degradome and transcriptome sequencing in hyperaccumulator Sedum alfredii uncovers a complex regulatory network and provides insights into cadmium phytoremediation. Plant Biotechnol. J..

[28] Pfaffl MW (2001). A new mathematical model for relative quantification in real-time RT–PCR. Nucl. Acids Res..

[29] Chen SS, Jing J, Han XJ, Zhang YX, Zhuo RY (2018). Identification, expression analysis of the Hsf family, and characterization of class A4 in Sedum alfredii Hance under Cadmium stress. Int. J. Mol. Sci..

[30] Finn RD, Clements J, Eddy SR (2011). HMMER web server: interactive sequence similarity searching. Nucl. Acids Res..

[31] Letunic I, Bork P (2018). 20 years of the SMART protein domain annotation resource. Nucl. Acids Res..

[32] Hua Z, Zou C, Shiu S-H, Vierstra RD (2011). Phylogenetic comparison of F-box (FBX) gene superfamily within the plant kingdom reveals divergent evolutionary histories indicative of genomic drift. PLoS ONE.

[33] Wang G-M (2016). F-box genes: genome-wide expansion, evolution and their contribution to pollen growth in pear (Pyrus bretschneideri). Plant Sci..

[34] Crooks GE, Hon G, Chandonia JM, Brenner SE (2004). WebLogo: a sequence logo generator. Genome Res..

[35] Jia Q (2017). Genome-wide analyses of the soybean F-box gene family in response to salt stress. Int. J. Mol. Sci..

[36] Magali L (2001). PlantCARE, a database of plant cis-acting regulatory elements and a portal to tools for in silico analysis of promoter sequences. Nucl. Acids Res..

[37] Paul S (2003). Cytoscape: a software environment for integrated models of biomolecular interaction networks. Genome Res..

[38] Samad L, Jason M, Yue D, Bader GD, Pico AR (2013). Cytoscape app store. Bioinform. (Oxf. Engl.).

[39] Cenciarelli C, Chiaur DS, Guardavaccaro D, Parks W, Pagano M (1999). Identification of a family of human F-box proteins. Curr. Biol..

[40] Kipreos ET, Pagano M (2000). The F-box protein family. Genome Biol..

[41] Mukesh Jain AN, Rita A, Pinky A, Swatismita R, Pooja S, Sanjay K, Akhilesh KT, Khurana JP (2007). F-box proteins in rice. Genome-wide analysis, classification, temporal and spatial gene expression during panicle and seed development, and regulation by light and abiotic stress. Plant Physiol..

[42] Chunru W, Huying L, Miaomiao T, Xiumei Y, Daqun L (2017). Research progress for F-box protein family function in Arabidopsis thaliana. Acta Bot. Boreali-Occident. Sin..

[43] Yanze L, Jia F, Yanli Y, Lu L, Jinguang H, Yang G, Changai W, Zheng C (2014). The SCF E3 ligase AtPP2-B11 plays a negative role in response to drought stress in arabidopsis. Plant Mol. Biol. Rep..

[44] Jia F (2015). SCF E3 ligase PP2-B11 plays a positive role in response to salt stress in Arabidopsis. J. Exp. Bot..

