# Supplementary material for: Genome-wide characterization of the hyperaccumulator Sedum alfredii F-box family under cadmium stress
Source: Sci Rep. 2021 Feb 4;11:3023. doi: 10.1038/s41598-021-82690-7 (PMC7862640; doi:10.1038/s41598-021-82690-7)
Supplement: Supplementary file 1 — Supplementary Information. [file 41598_2021_82690_MOESM1_ESM.pdf]

Supporting Information

Genome-wide characterization of the hyperaccumulator *Sedum alfredii* F-box family under cadmium stress

Zhuang Zhang[1,2,3], Xiaojiao Han[2,3], Wen Liu[1], Wenmin Qiu[2,3], Longhua Wu[2,4], Miao Yu[2,3], Xuelong Qiu[5], Zhengquan He[1*], Renying Zhuo[2,3*]

1. Key Laboratory of Three Gorges Regional Plant Genetic & Germplasm Enhancement (CTGU)/ Biotechnology Research Center, China Three Gorges University, Yichang 443002, Hubei, China

2. State Key Laboratory of Tree Genetics and Breeding, Chinese Academy of Forestry, Beijing, China

3. Key Laboratory of Tree Breeding of Zhejiang Province, the Research Institute of Subtropical of Forestry,

Chinese Academy of Forestry, Hangzhou, Zhejiang, China

4. Key Laboratory of Soil Environment and Pollution Remediation, Institute of Soil Science, Chinese Academy of Sciences, Nanjing 210008, China.

5. Agricultural Technology Extension Center of Fuyang District, Hangzhou, Zhejiang, China

# These authors contributed equally to the work.

* Authors for correspondence:

Zhengquan He

Tel: 86-7176397188

Email: zhq_he@163.com

Renying Zhuo

Tel: 86-571-63311860

Email: zhuory@gmail.com

Table S1: Nucleic acid sequences and the encoded amino acid sequences of the SaFbox family members.

It has been uploaded as an excel spread sheet

Table S2: CDS length, number AA, PI and Molecular weight were analysis by different softwave or online website.

It has been uploaded as an excel spread sheet

Table S3: All of SaFbox' conserved domain were predicted by online website.

It has been uploaded as an excel spread sheet

Table S4: The sequence of SaFbox' promoter.

It has been uploaded as an excel spread sheet

Table S5: List of hub genes.

It has been uploaded as an excel spread sheet

Table S6: Expression of SaFbox in differernt tissues at stage under Cd stress.
